# Differential 28-Days Cyclic Modulation of Affective Intensity in Female and Male Participants via Social Media

**DOI:** 10.3389/fnint.2019.00005

**Published:** 2019-02-19

**Authors:** Lucila Gallino, Facundo Carrillo, Guillermo A. Cecchi

**Affiliations:** ^1^Immunopharmacology Lab, IQUIBICEN, Buenos Aires University, Buenos Aires, Argentina; ^2^Applied Artificial Intelligence Lab, ICC, CONICET, Buenos Aires, Argentina; ^3^Computational Biology Center, T.J. Watson Research Center, IBM, New York, NY, United States

**Keywords:** natural language processing, computational linguistic, emotional regulation, menstrual cycle, 28 days cycle, social media

## Abstract

The menstrual cycle affects many aspects of female physiology, from the immune system to behavioral and emotional regulation. It is unclear however if these physiological changes are reflected in everyday, naturalistic language production, and moreover whether these putative effects can be consistently quantified. Using a novel approach based on social networks, we characterized linguistic expression differences in female and male volunteers over the course of several months, while having no physiological or reported information of the female participants' menstrual cycles. We used a simple algorithm to quantify the linguistic affect intensity of 418 (184 females and 234 males) subjects using their social networks production and found a 7-day modulatory cycle of affect intensity that corresponds to labor-week fluctuations, with no significant difference by biological sex, and a 28-day cycle over which females are significantly different than males. Our results are consistent with the hypothesis that the menstrual cycle modulates affective features of naturalistic linguistic production.

## 1. Introduction

Hormonal regulation affects multiple facets of animal behavior, and is expressed in humans along two main dimensions: development (Silk et al., 2009) and sex (Chiazze et al., 1968). The menstrual cycle (MC), which typically occurs in human females every 28 days (Chiazze et al., 1968; Gizzo et al., 2015; Reed and Carr, 2015; Santoro et al., 2017; Xiao et al., 2017), results from a hormonal regulation whose complexity is matched by its paramount evolutionary role. MC phase affects many aspects of physiological function, including the regulation of the immune system (Pehlivanoglu et al., 2001), appetite (Dye and Blundell, 1997), responses to exercise (Pivarnik et al., 1992; de Jonge, 2003) and pain (Houghton et al., 2002; Powell-Boone et al., 2005). These phases also regulate cognitive, behavioral and emotional functions such as memory (Hampson, 1990; Postma et al., 1999), decision making (Meadowcroft and Zillmann, 1987; Chavanne and Gallup, 1998), sexual preferences (Backstrom et al., 1983; Bancroft et al., 1983; Sanders et al., 1983; Harvey, 1987), sexual frequency (Udry and Morris, 1968) and mood (Moos et al., 1969; Parlee, 1973; Romans et al., 2012; Wu et al., 2014).

It is expected, therefore, that the profound effects of MC would include one of the most distinctive and complex of human features, namely language production. Specifically, we aimed to test the hypothesis that the cyclic regulation of the MC leaves an imprint in the linguistic production of females engaged in social media, strong enough to be discriminated from that of matching male participants. Previous studies have used language production to characterize changes in mental state elicited by psychoactive drug intake and psychosis, among others (Bedi et al., 2014, 2015; García et al., 2016; Mota et al., 2016; Carrillo, 2017; Carrillo et al., 2018; Corcoran et al., 2018). Massive textual content in social networks has been used to identify abrupt changes in semantic space of concepts caused by salient events (Carrillo et al., 2015), as a possible indicator of depression using subject's Facebook public information (De Choudhury et al., 2013), or more specifically to characterize and predict postpartum depression (De Choudhury et al., 2014). To our knowledge, however, the precise effects of MC on language production has not been studied with analytic methods.

There are many ways to quantify the relationship between language and MC. The most parsimonious approach would be to do so in a controlled environment, obtaining prospective samples of the participants' speech and MC data, typically length of their MC, the phase, etc., and eventually perform a clinical intervention to measure exactly the MC state and hormones level. This approach, however, is of limited scalability and moreover introduces additional experimental effects in the responses due to the awareness of the participants. We decided to follow an alternative approach, posing limitations as well as significant advantages, by tapping into social media activity. In particular, we performed a large retrospective analysis of Twitter feeds from a population of self-described female and male participants, aiming to quantify changes of affective content in linguistic productions consistent with the expected modulation over the menstrual cycle. We reasoned that affective content would be the most likely effect to be detectable in the constrained setting of social media activity, given that many of the demonstrated regulatory effects of the MC mentioned above do contain an explicit or implicit affective valence component.

## 2. Methods

### 2.1. Subjects and Timeline Description

To address our main hypothesis we collected data from 2,000 attendants at a conference. We asked them for their Twitter username, age, native language, and biological sex. Then, we obtained the tweets (the name of every message on Twitter, the biggest microbbologin platform) using the Twitter API http://www.webcitation.org/6xWYUH6jQ following the Terms & Conditions of Twitter.

After downloading all the tweets, we deleted the Twitter username and all mention of other Twitter users in every message to guarantee the anonymity of the data

As we needed to get dense data to address our hypothesis, we excluded all participants that did not present enough data, we defined the minimum data required as a production of 2 messages per day in the last continues interval of 280 days production. We also excluded participants that did not inform biological sex (or they wrote down something different of female/male) and those their native language was not Spanish.

After excluding participants, we obtained a sample of 418 voluntary participants (184 females and 234 males, 18–40 years old). The average age was 26.8 years, with a standard deviation of 5.9 years (male 27.39 ± 5.86, female 26.22 ± 5.67, not statistically different).

During the 280 days periods we recollected the messages, the average production was 7.31 tweets per day, with a standard deviation of 2.22. The volume of tweets is comparable, if not larger, than what was used in similar recent work, see for example (Golder and Macy, 2011).

The procedures of the experiments described here were approved by the ethics committee of CEMIC (Centro de Educación Médica e Investigaciones Clinicas Norberto Quirno). The participants read and accepted the written informed consent when they completed the data form with their information.

### 2.2. Affect Intensity Measure

The affect intensity algorithm (AI) is a technique for quantifying the affectivity of a text. The AI measures the ratio of high affect words independent of the polarity of the emotion, i.e., it only estimates the intensity of the emotion and not its positive or negative valence; e.g., “hate” and “love” are equally intense. For this, we used a list of positive affect and negative affect words from Rıos and Gravano (2013). In that work, Spanish DAL, the authors repeated the experiment of Whissell et al. (1986) in Spanish with a lexicon formed by more than 2,500 words manually rated along the same three dimensions of the original work: pleasantness, activation and imagery. We used pleasantness to define positive affect (PA) and negative affect (NA) lists. Words with pleasantness value smaller than 20% of all words in the pleasantness scale were considered Negative Affect words. Conversely, words with pleasantness value greater than 80% of all words in the pleasantness scale were considered Positive Affect words. Then, we defined the list of high affect intensity words as the union of both sets (PA and NA) of words.

With this set of words, we defined the affect intensity score of a sentence as the rate of words in the sentence that are included in the list. Positive and Negative Affect scores were defined as the rate of positive and negative words included in the sentence, respectively. For example, the sentence “This is a beautiful day” has only 5 words, but just one belongs to the high affect list: (beautiful). Then, AI is 0.2, the Positive Affect score is 0.2 and the Negative Affect score is 0.

As defined, AI scores may be calculated for each individual sentence. To evaluate the AI score of a text, we split it into sentences and calculate AI scores for each of them and summarize statistic using the mean and standard deviation of the sentence AI score series. When we applied this method to evaluate the AI of a tweet, we considered every tweet as a simple sentence, we cleaned the text and we used only letters and spaces characters converting every character in lower case. We did not use others characters as emojis, at-sign (@) and hashtag (#).

### 2.3. Experimental Design

We asked, following our main hypothesis, if there are any 28 days cyclic fluctuations in the affective expression on biological sex female participants. We propose 28 days as an average menstrual cycle length because we did not collect either information about the length of the menstrual cycle nor the phase of the menstrual cycle. Indeed we did not ask if the female participants were pregnant at any moment of the 280 days interval we used.

Therefore, as we could not synchronize every female participants with the other in function of their menstrual cycle, we used the autocorrelation functions as a tool to compare between biological sex groups. The autocorrelation function is the cross-correlation of a signal with itself at different points in time, or lags. For example, if we have a signal with one value per day, when we compute the autocorrelation function of this signal, the value of the function for *lag*=0 is 1 because this value corresponds with to correlation of the signal itself, but if we compute the value of the function for the *lag*=1, we get the correlation between the signal in 1 day and the signal the next day. In our work, we used autocorrelation functions to quantify the length of the different repetitive patterns and identify the occurrence of periodical fluctuations. The autocorrelation function for the *lag*=*n* quantifies the effect of repetitive patterns of length *n*.

**Figure 1:**
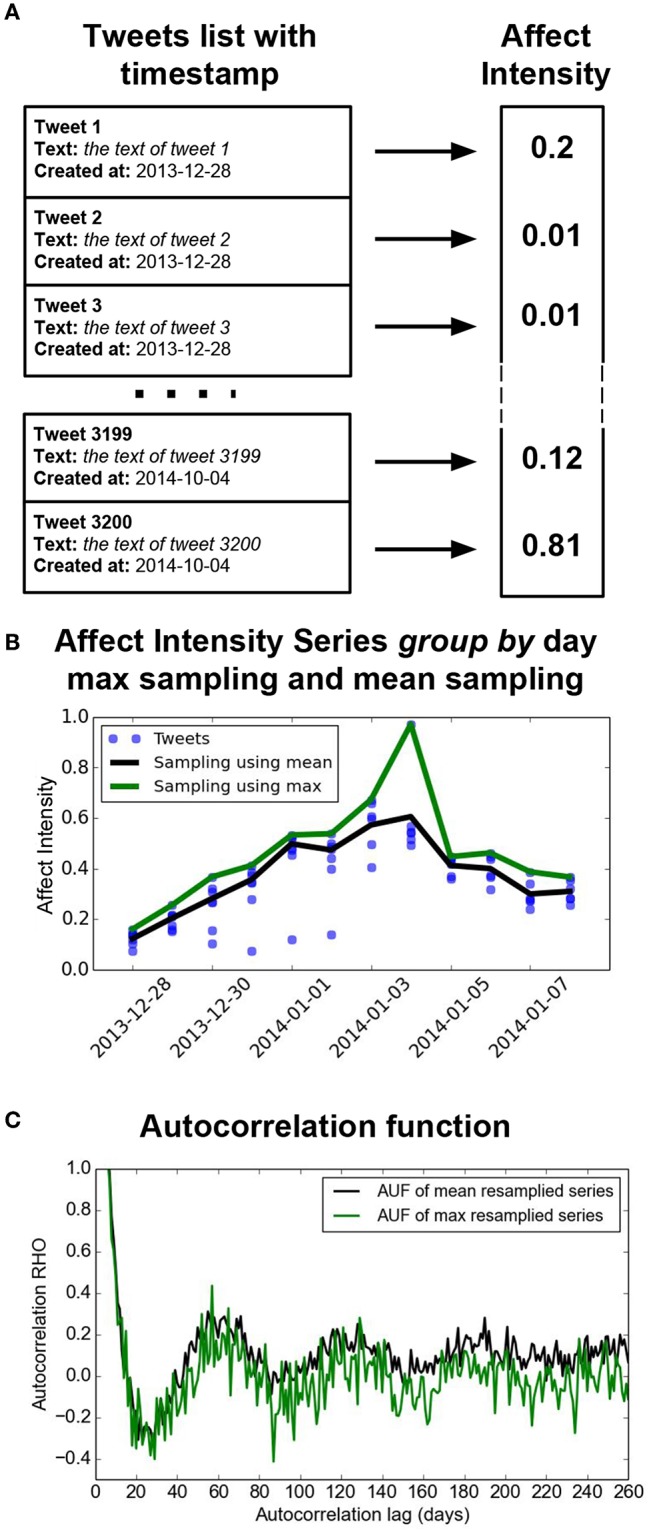
Experimental Pipeline: This figure shows an example of the process that we implemented for every subject. The fist step **(A)** is the download process, where we get the last 3,200 tweets of a particular subject. The second step **(B)** is to compute the affect intensity value for every subject. With this list of 3,200 affect intensity values and there timestamps we resampled by computing the maximum (green line) and mean (black line) values to represent each day. With these two computed timeseries we calculated the autocorrelation as a function of the day (the lag) **(C)**.

The analytic pipeline is the following. For each subject: (1) We downloaded the last 3,200 tweets (for limitation of the API we could not download more than 3,200 tweets). (2) We computed the AI score of each tweet. (3) We computed for each day, two summary statistics: max and mean. (4) For the resulting time series (with the AI score of each day as a point) we computed the autocorrelation functions based on mean and max. Once the autocorrelation function for each subject was computed, we ran statistics to identify at what lag (in days) these functions differed by biological sex. Figure 1 shows the pipeline.

## 3. Results

### 3.1. Affect Intensity Validation

**Figure 2:**
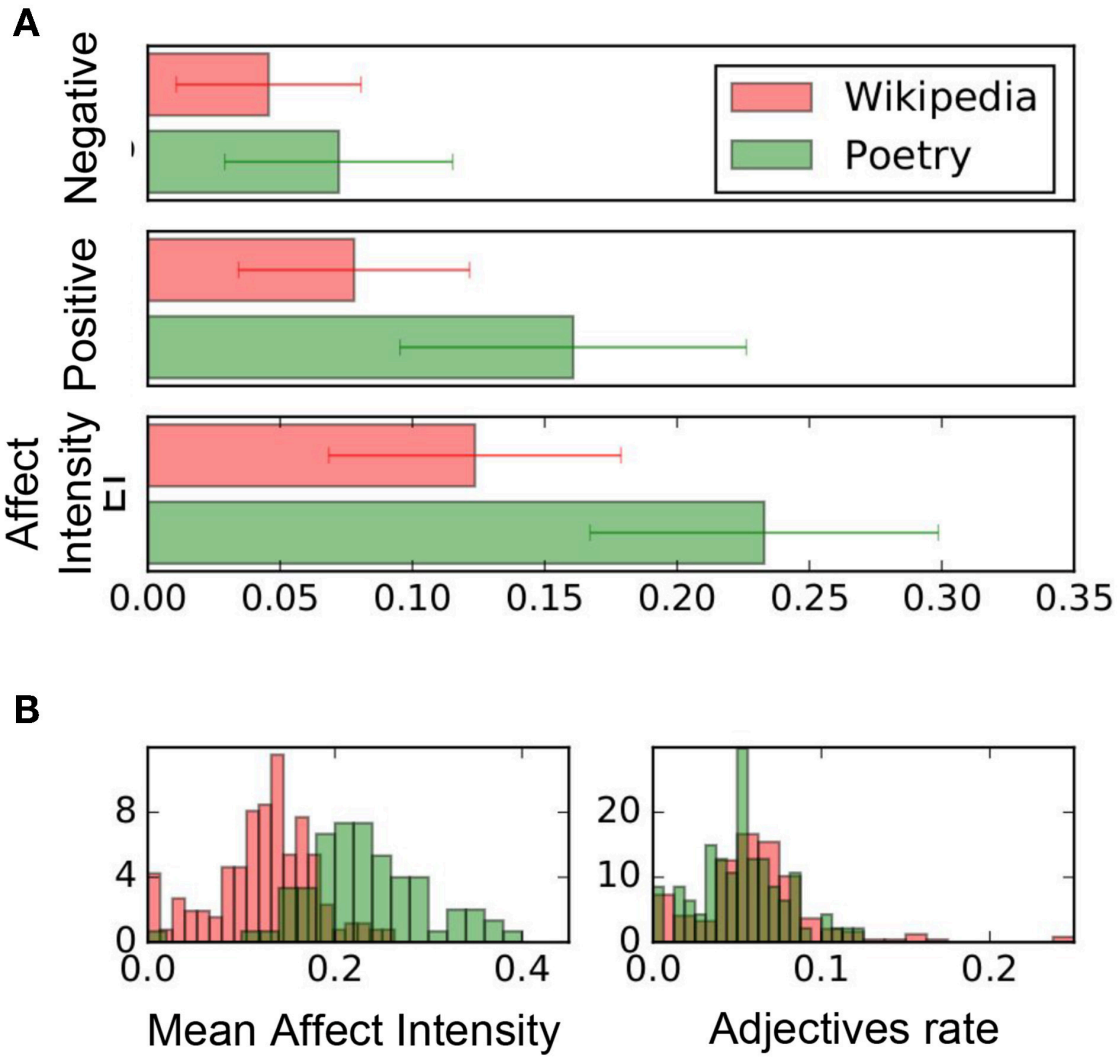
Positive and Negative Affect and Affect Intensity scores for Wikipedia and poetry. Each bar reports the mean value and the standard deviation. **(A)** Shows the expected increase of NA, PA, and AI in poetry over Wikipedia (*p* − *value* < 0.001). **(B)** Shows the distribution of mean AI the rate of adjective use in each corpus. The difference in AI is statistically significant (*p* − *value* < 0.001), while the adjective rate is not.

Before we tackled our main hypothesis we ran a control experiment of the Affect Intensity measure. We tested the difference on the score between two different corpus. We used Wikipedia (200 articles chosen randomly) and in poetry (100 poems from the site best-poems.net). Wikipedia texts had a lower average scores in Positive Affect (PA), Negative Affect (NA) and AI than the poetry corpus and all differences were significantly (*P* < 0.001). Figure 2 shows these values. This result reflects the affectivity of the words, and not merely the number of adjectives, since the fraction of adjectives did not change in the two datasets (*P* = 0.31).

### 3.2. Effects of Menstrual Cycle in AI Scores Time Series

For each subject, we calculated two different time series from tweets: one averaging AI scores across all tweets in a day, and the other considering the tweet with maximal AI score in that day.

**Figure 3:**
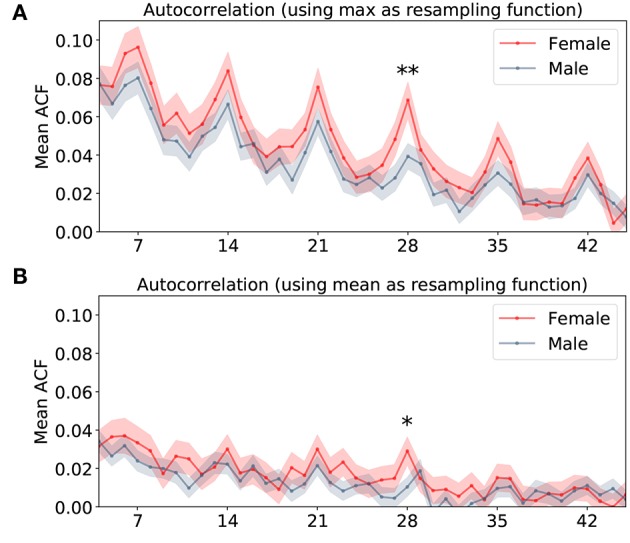
Mean and standard error of the autocorrelation functions of the AI score timelines grouped by biological sex. Red line represents female time series and blue one represents male time series. **(A)** Shows the ACF derivate from the AI score series using max as day-sampling function. The double cross marks the only lag (28 days) where both groups are significantly different (*p* − *value* = 0.00853). **(B)** Shows the ACF derivate from the AI series using mean as day-sampling function. The cross marks the only lag (28 days) where both groups are significantly different (*p* − *value* = 0.02605).

As our goal was to study language modulations during the menstrual cycle, we needed to test whether there are significant differences between males and females, i.e., whether females have as a group a higher or lower mean than males. We did not find any statistical difference between the groups in any of the two time series (student test *P* = 0.20 for mean resampling and *P* = 0.31 for max resampling). This suggests that there is not biological sex difference between the use of affect intensity in language, assuming it has a stationary distribution. However, the menstrual cycle modulation should be expressed as differences in the temporal structure between male and female tweets. For this, we computed the autocorrelation function (ACF), which estimates the similarity in AI scores for different time lags, and grouped the data averaging the ACFs for males and females (Figure 3).

The ACF showed two clear patterns. First a long-term decrease, which simply indicates that as time goes on, the AI scores slowly decorrelate. Second, weekly peaks above this general trend, which indicates that AI scores tend to show similar patterns in the same days of the week. Above and beyond these two findings, our main hypothesis is that females should show, compared to males, a different behavior around a lag of 28 days (the average of female menstrual cycle Chiazze et al., 1968; Gizzo et al., 2015; Reed and Carr, 2015; Santoro et al., 2017; Xiao et al., 2017).

**Figure 4:**
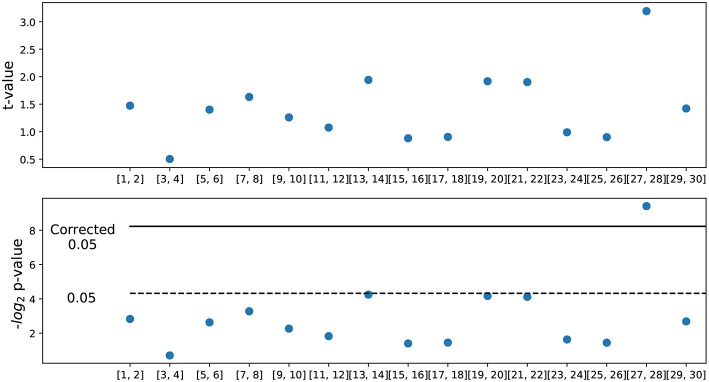
Statistical significance of differences in autocorrelation. **(Upper panel)** *t*-value of the comparison between two consecutive days for males and females; positive values indicate female higher than male. **(Lower panel)** Statistical significance in −log_2_(*p*) units. The dashed line corresponds to *p* = 0.05 and the solid line to the correction for 15 comparisons. Observe that the lags at 27 and 28 days correspond to the only statistically significant difference between males and females.

The ACF for the female was slightly above that for males throughout. A statistical comparison of male and female ACFs showed that the only significant difference between the two groups (for the two ACF versions) was for the lag day 28. This was the case for both summary statistics (mean and max): for max(AI), student test *p*-value = 0.00853, Cohen effect size 0.288, for mean(AI) it was *p*-value = 0.02605, Cohen effect size 0.244). While the difference in max(AI) is significant for a single comparison at a 0.05 threshold, it is not if we account for multiple comparisons assuming 30 comparisons (*p* = 0.0016). In order to obtain a corrected significance, we pooled data from two consecutive days instead of single ones, so that we compare ACF for lags 27 and 28 against the other lags. In this case, the test yields significance with *p* = 0.0015, which survives correction for 15 (and even 30) comparisons. This is shown in Figure 4, representing the *t*-value and *p*-value for each of the pairs of days considered. This shows that above and beyond weekly fluctuations, the similarity of affect intensity in lags of 28 days is greater for females than for males, a direct prediction of the hypothesis that the MC modulates affective intensity.

We next investigated whether this increase in the similarity in 28-day lag for AI scores is driven by a specific bias in affect valence, be it positive or negative. To this aim, we repeated the same analysis distinguishing between affect of positive and negative valence. None of these series individually (positive and negative) show any significant difference between males and females.

## 4. Discussion

We hypothesized that the affective content of linguistic production is modulated by the menstrual cycle. To address this, we designed a non-intrusive experimental paradigm that allowed us to collect more than 1.3 million messages in a continuous time period from female and male Twitter users. We implemented a simple natural language processing algorithm that quantifies the rate of the use of high affectivity words as an Affect Intensity score. We found that female participants presented a higher autocorrelation than male participants for 28 days cycles, which means that for females the AI scores of a day *T* are more similar to that of day *T* + 28 than for males. We understand that the most probable explanation of this result is that the MC is partially operating in females because it would be the only variable that different between both groups.

The algorithm proposed here has some clear limitations, well-known in computational studies of affective valence. For instance, a sentence like “The house is not so bad” is considered negative because it contains the word “bad,” but anyone with basic knowledge of English would conclude that the valence of this sentence is positive. This can be remediated using more complex, contextual models of sentiment analysis, see e.g., Socher et al. (2013). However, this degree of sophistication was not necessary to solve the main objective of this work. Twitter has a strong limitation in the length of messages that users can write, and even though emojis provide an additional expressivity dimension, it is quite limited and we decided to ignore it. However, there are many other social networks more oriented to other types of production. It may be possible, for example, to study changes in type of posted photos in Instagram, by automatically quantifying features in images.

It is important to emphasize that this result does not mean that female participants show an overall hightened (or lowered for that matter) affective intensity than male participants as expressed in their tweets. As mentioned in the Results section, we could not discriminate between males and females based on the stationary distribution of score values. The differentiation can be seen only in the correlation of females scores estimated 28 days apart. We do not have sufficiently detailed data to answer the question as to what happens with affective intensity within the menstrual cycle, for which we would need to record time the precise phase for each individual in order to reveal more fine-grained correlations with the various hormonal dynamics, but this would require an experimental setting with more active involvement from female participants. A parimonious interpretation of our result, nevertheless, is that individual females are affected by the menstrual cycle in different but consistent ways. That is, one particular female may express a hightened intensity during the initial phase to then experience a decrease, while for another it may be the opposite, but in both cases these patterns should be consistent over consecutive cycles.

There are some results in the literature on mood changes during pregnancy and the puerperal (Elliott et al., 1983; Smith et al., 1990; Ross et al., 2004). These two stages presented different dynamics in the concentration of many hormones involved in MC. Moreover, there is evidence of a relationship between the premenstrual syndrome and postpartum depression, both conditions also related to changes in the endocrine system (Hammarbäck et al., 1989; Pearlstein, 1995; Soares and Zitek, 2008). In the same vein, our results suggest that the fluctuations we observed should not be seen in females post menopause or similarly at ages before the onset of the menstrual cycle. Including this type of additional information should provide for a highly detailed characterization of the relationship between hormonal production and emotional regulation.

Our result is consistent with previous findings showing the involvement of hormones in different cognitive functions. Collecting this information could be useful to understand how hormones could be part in medical interventions or to understand better the side effects of therapies that have already used hormones to treat patients. Moreover, in combination with additional digitaly-delivered probes it may be possible to gather information regarding the relation between emotional states and risk-taking and decision-making behaviors, memory processing and other functions not so obviously expressed in short language samples, relevant also for other axes beyond gender, for instance age and cultural and socio-economic background.

Recent studies on effective prognosis of psychotic outbreak based on computational linguistic analysis of transcribed interviews (Bedi et al., 2015; Corcoran et al., 2018) suggest that, by combining these methods with the permanent access to human production in social networks, automated monitor systems could prevent extreme events and warn subjects to visit a specialist. Preliminary results already show that this may be the case for the prediction of psychosis relapse (Birnbaum et al., 2018), in the context of which our study could applied to explore interactions between mood and relapse propensity in males and females, as well as hormonal regulation and relapse in females. Other mental health conditions, prominently major depressive disorder, could equally benefit from the approach proposed here.

## Author Contributions

FC and GC analyzed the data. LG and FC conceived the analysis. LG set up the biological framework. FC coded the scripts to get the data. All authors wrote the final version of the manuscript.

### Conflict of Interest Statement

The authors declare that the research was conducted in the absence of any commercial or financial relationships that could be construed as a potential conflict of interest.
